# Construction of a risk prediction model for premature rupture of membranes based on vaginal microecology and systemic inflammation: a prospective cohort study

**DOI:** 10.3389/fcimb.2026.1777431

**Published:** 2026-03-19

**Authors:** Xiaoyan Xu, Yuance Xu, Xuehui Zhen, Jianguang Zhang, Chenyang Li

**Affiliations:** 1 Jilin Women and Children Health Hospital, Jilin Provincial Obstetric Quality Control Center, Changchun, China; 2 Jilin Province Institute of Population Life Sciences and Technology, Jilin Province Reproductive Health Care Hospital, Changchun, China

**Keywords:** group B *Streptococcus*, internal validation, nomogram, pregnancy outcome, pregnant woman, premature rupture of membranes, reproductive tract microenvironment

## Abstract

**Objective:**

To investigate whether vaginal microecological imbalance and group B *Streptococcus* (GBS) colonization are correlated with premature rupture of membranes (PROM) by activating the maternal systemic inflammatory response and to construct an individualized risk prediction model.

**Method:**

This study was a prospective single-center cohort study. A total of 162 singleton pregnant women at 28–37 weeks of gestation who were admitted to Jilin Provincial Maternal and Child Health Hospital from January 2023 to June 2024 were included. Among them, 81 cases were in the PROM group, and 81 cases were in the matched control group. Vaginal secretions were collected for microbiological testing (Nugent score, H_2_O_2_, and leukocyte esterase), and GBS colonization was detected by the constant temperature amplification method; serum levels of CRP, PCT, IL-6, and IL-10 were determined by electrochemiluminescence and chemiluminescence methods. LASSO-penalized multivariable logistic regression analysis was used to identify independent risk factors, and a nomogram model was constructed with internal validation by bootstrap resampling (1,000 iterations).

**Result:**

The GBS colonization rate in the PROM group was significantly higher (16.0% vs. 3.7%, aOR = 4.87, 95% CI 1.89–12.56). The incidence rates of abnormal vaginal microscopic evaluation (grades III–IV), H_2_O_2_ depletion, and leukocyte esterase positivity were all significantly higher than those in the control group (*P* < 0.01). The ratio of IL-6/IL-10 in the PROM group was significantly increased (*P* < 0.001). The multivariate model showed that GBS colonization, IL-6 >12 pg/mL, and H_2_O_2_ depletion were independent risk factors. The nomogram model constructed based on these indicators had an apparent AUC of 0.89 (95% CI 0.83–0.95) and a bootstrap-corrected AUC of 0.86 (95% CI 0.80–0.92) and excellent calibration (Brier score 0.158, Hosmer–Lemeshow *P* = 0.423).

**Conclusion:**

GBS colonization is associated with a higher incidence of PROM by inducing vaginal microecological imbalance and systemic inflammatory responses. The “microecology–inflammation” nomogram model we have developed shows potential for individualized prediction of the risk of PROM pending external validation, providing a basis for early intervention.

## 1 Introduction

Premature rupture of membranes (PROM) refers to the spontaneous rupture of the fetal membranes before 37 weeks of gestation, which is the primary cause of preterm birth, accounting for 30%–40% of all preterm births. It is also closely associated with severe complications such as neonatal respiratory distress syndrome, necrotizing enterocolitis, cerebral palsy, maternal chorioamnionitis, and puerperal infection (Skupski, 2019; Dayal et al., 2025). Although international guidelines generally recommend administering antibiotics combined with glucocorticoids to patients with PROM to promote fetal lung maturation, the incidence has not significantly decreased in the past decade, suggesting that there is still a significant gap in our understanding of the etiology of PROM (Mercer et al., 1997; Medina and Hill, 2006; Middleton et al., 2017). The traditional view holds that upward infection triggers a local inflammatory cascade reaction, activating proteolytic enzymes and degrading the extracellular matrix (ECM) of fetal membranes, which is the core mechanism of membrane rupture (Menon and Richardson, 2017; Lin et al., 2024); however, based on culture-based pathogen detection, only 30%–40% of PROM cases can detect clear pathogens, suggesting that “latent infection” or “microbial imbalance” may be an earlier driving event (Romero et al., 2015). In recent years, vaginal microbiome studies have found that the diversity of vaginal microbiota in PROM patients increases, while the relative abundance of *Lactobacillus crispatus* decreases, and aerobic bacteria (such as *Escherichia coli*), anaerobic bacteria (such as *Gardnerella vaginalis*), and opportunistic pathogens (such as group B *Streptococcus*, GBS) significantly increase (Zhang et al., 2017).

At the local level, GBS colonization triggers a cascade of inflammatory events. GBS surface proteins, such as alpha-like protein (Alp) family and pilus proteins, facilitate adherence to vaginal epithelial cells and subsequent invasion. The recognition of GBS pathogen-associated molecular patterns (PAMPs) by pattern recognition receptors (PRRs), particularly TLR2 and TLR4 on epithelial cells and resident immune cells, initiates the NF-κB signaling pathway. This leads to the production of pro-inflammatory cytokines (IL-1β, IL-6, TNF-α) and chemokines (IL-8, MCP-1), promoting neutrophil recruitment and the characteristic leukocyte esterase positivity observed in clinical assessments. The local inflammatory microenvironment, characterized by decreased H_2_O_2_-producing lactobacilli, further facilitates GBS proliferation through loss of competitive exclusion and reduced bacteriocin production.

GBS, as a Gram-positive opportunistic pathogen, has a colonization rate of 10%–30% in the pregnant woman’s vagina, varying by region, race, and detection method (Goel et al., 2020); previous studies have mostly focused on the association between GBS colonization and neonatal early-onset sepsis (EOS) but paid insufficient attention to its causal role in the occurrence of PROM (Johri et al., 2006). Recent *in vitro* and animal experiments have shown that GBS can secrete hyaluronidase and C5a peptidase, directly degrading the components of the fetal membrane matrix; form biofilms, enhancing immune evasion; and simultaneously activate the Toll-like receptor 2/4 (TLR2/4)–NLRP3 inflammatory pathway, inducing the release of pro-inflammatory factors such as IL-1β, IL-6, and TNF-α, thereby upregulating the expression of matrix metalloproteinases (MMP-2/MMP-9) and degrading type IV collagen and elastin (McCutcheon et al., 2021; Coleman et al., 2023).

The translocation of inflammatory mediators from the local site to systemic circulation is mediated by cytokine spillover and activated immune cell migration. The observed elevation in serum IL-6 reflects not only local production but also hepatic synthesis in response to systemic inflammatory stimuli. Importantly, the IL-6/IL-10 ratio serves as a barometer of the balance between pro-inflammatory and regulatory responses. A ratio >2.0 in our study suggests a tilt toward inflammatory dominance, which may reflect either inadequate regulatory control or compensatory exhaustion, both of which compromise fetal membrane integrity through activation of MMPs and prostaglandin synthesis.

Regarding the host response, the role of anti-inflammatory factor IL-10 is still controversial: it can inhibit Th1-type immune responses, limit tissue damage, or may reflect that the body is in an “immune exhaustion” state, unable to effectively eliminate pathogens (Puliti et al., 2002; Ouyang et al., 2011); simply detecting the absolute levels of IL-6 or IL-10 may not be sufficient to depict the dynamic balance of host–pathogen interaction, and the IL-6/IL-10 ratio may better reflect the deviation direction of the “inflammation-inhibition” axis, thereby indicating the risk of PROM (Ouyang et al., 2011). The commonly used vaginal microscopic evaluation, H_2_O_2_ content, and leukocyte esterase detection are simple to operate, but their independent value in predicting PROM has not been systematically verified; we speculate that GBS colonization induces vaginal microecological imbalance (H_2_O_2_ decrease, abnormal microscopic evaluation) and activates systemic inflammatory responses (increased IL-6/IL-10 ratio), ultimately increasing the risk of PROM (Coleman et al., 2023).

This study aims to 1) verify the association between GBS colonization and vaginal microecological indicators (microscopic evaluation, H_2_O_2_, leukocyte esterase) as well as systemic inflammatory indicators (CRP, PCT, IL-6, IL-10), 2) explore the independent contribution and interaction of these indicators in the occurrence of PROM, and 3) construct and evaluate a “microecology–inflammation” nomogram model based on the data of this cohort for its discrimination and calibration, laying the foundation for subsequent external validation. This study is expected to clarify the associative pathway of GBS colonization in the occurrence of PROM and provide a theoretical basis and population foundation for future targeted microecological interventions (such as probiotics, GBS vaccines).

## 2 Materials and methods

### 2.1 General information

From January 2023 to June 2024, 81 pregnant women with PROM during the late pregnancy period who visited our hospital were selected as the research group, while 81 healthy pregnant women during the late pregnancy period during the same period were selected as the control group. This study was conducted in accordance with the Declaration of Helsinki and approved by the Ethics Committee of Jilin Women and Children Health Hospital (Approval No. 2025004). All participants provided written informed consent. The inclusion criteria for cases were as follows: 1) singleton pregnancy; 2) good cognitive ability, able to understand and cooperate with the examination; 3) pregnant women who completed relevant examinations during regular prenatal check-ups and delivered; 4) patients informed of the research content and voluntarily signed a written informed consent form; and 5) complete clinical data. The exclusion criteria include 1) multiple gestation in the current pregnancy; 2) severe maternal diseases (cardiac, renal, hepatic, endocrine, autoimmune, or uncontrolled infectious disorders); 3) history of sexual activity or vaginal medication within 3 days before the examination; 4) using antibiotics, immunosuppressants, or sedatives and other drugs; and 5) unable to cooperate with follow-up. For PROM diagnosis, rupture of membranes before labor is called premature rupture of membranes. Vaginal fluid was collected for smear operation. If crystalline substances were observed under the microscope in the vaginal fluid, and the pH value of the vaginal fluid was greater than or equal to 6.5, PROM was considered. Normal vaginal pH in late pregnancy is 3.8–4.5. A pH ≥6.5 was used as an ancillary diagnostic criterion for PROM, following the 2021 Chinese guideline and our hospital’s protocol.

### 2.2 Method

#### 2.2.1 Basic information

We collected the age, gestational weeks, body mass index, and number of pregnancies of pregnant women and carefully recorded whether they have concurrent conditions such as hypertension, diabetes, hypothyroidism, etc.

#### 2.2.2 Pathogenic bacteria and microenvironment detection

We analyzed vaginal secretions using the Vaginitis Combined Test Kit (dry chemistry method, product code: LT-V200, Li Tou Biotechnology Co., Ltd., Zhuhai, China) to assess the microenvironment of the reproductive tract. The test utilizes a horseradish peroxidase-catalyzed chromogenic reaction. H_2_O_2_ depletion was defined as values below the kit threshold of 2 μmol/L, indicating reduced *Lactobacillus* peroxidase activity and compromised vaginal defense. Leukocyte esterase positivity was determined by a colorimetric scale ≥1 grade (light yellow) according to the kit instructions. We detected vaginal trichomonas using a physiological saline smear to determine if trichomonas vaginitis was present. We treated the sample with 10% potassium hydroxide solution and observed under a microscope for the presence of pseudo-hyphae and budding spores to detect vaginal *Candida*. Bacterial vaginosis was diagnosed based on the Amsel clinical diagnostic criteria (homogeneous, thin, grayish-white vaginal secretions; vaginal pH > 4.5; positive amine test; positive clue cells in three out of four items). In addition, we conducted vaginal microscopic evaluation using Gram staining to prepare slides of vaginal secretion samples; observed the distribution density of epithelial cells, inflammatory cells (white blood cells), lactobacilli, and other cocci under an optical microscope; and classified vaginal microscopic evaluation into four grades according to the standards outlined in the “National Clinical Laboratory Operating Procedures” as follows: grade I—microscopic observation shows a large number of lactobacilli and intact epithelial cells, no pathogenic bacteria, or inflammatory cells, with a clear background; grade II—the number of lactobacilli and epithelial cells is moderate, accompanied by a small amount of inflammatory cells and opportunistic pathogens, which is a normal physiological manifestation; grade III—the number of lactobacilli and epithelial cells is reduced, and the number of opportunistic pathogens and inflammatory cells is increased; and grade IV—lactobacilli are completely absent, and the field is filled with pus cells and a large number of opportunistic pathogens. The pH value, white blood cell esterase activity, and hydrogen peroxide concentration of vaginal secretions were all detected using an automatic analyzer, and all operating procedures followed the manufacturer’s standardized testing. White blood cell esterase was detected using white blood cell esterase test strips by immersing the strips in the collected vaginal secretion sample and reading the result according to the manufacturer’s instructions. If the color changes, the white blood cell esterase was determined as positive according to the colorimetric card. Hydrogen peroxide levels in vaginal secretions were detected using hydrogen peroxide test strips, following the manufacturer’s instructions. We recorded values below the kit threshold as H_2_O_2_ depletion (below the normal range), which is determined as an abnormal hydrogen peroxide level. A value below the kit threshold was recorded as H_2_O_2_ depletion, indicating reduced *Lactobacillus* peroxidase activity.

We performed GBS detection. Before sampling, the external genitalia were routinely disinfected, and a secretion sample was obtained from the lower one-third of the vagina. The collected vaginal secretion was evaluated for GBS using the loop-mediated isothermal amplification method (Group B *Streptococcus* Nucleic Acid Detection Kit, catalog number: MF-GBS-001, Jilin Maofu Biotechnology Co., Ltd., Changchun, China), targeting the cfb gene encoding CAMP factor, with a detection limit of 10² CFU/mL, and the corresponding quantitative test was carried out according to the relevant steps of the kit.

#### 2.2.3 Inflammatory marker detection

Five milliliters of fasting venous blood was collected from pregnant women. After allowing to stand for 30 min, it was centrifuged at 3,000 revolutions per minute for 15 min to separate the serum. The serum CRP level was detected using the electrochemiluminescence method with a Cobas e601 analyzer (Roche Diagnostics, catalog 11821629, Sichuan, China). We determined PCT using the electrochemiluminescence method, with a PCT detection reagent (Sichuan Woven Biotechnology Co., Ltd., WW-PCT-100, Sichuan, China), following the operation steps provided in the manufacturer’s manual. Serum IL-6 and IL-10 levels were detected using the chemiluminescence method, with the iFlash 3000 platform and test kits (Sichuan Woven Biotechnology Co., Ltd., WW-IL6–100 and WW-IL10-100), according to the operation steps in the kit manual.

#### 2.2.4 Pregnancy outcomes

The following outcomes were recorded: cesarean section rate of pregnant women, postpartum hemorrhage of the mother, chorioamnionitis, fetal distress, puerperal infection, neonatal asphyxia, premature birth, etc.

### 2.3 Statistical method

The data processing in this study was conducted using SPSS 27.0 and R version 4.3.1 software. Normality was assessed using the Shapiro–Wilk test. Normally distributed continuous variables (age, BMI, gestational weeks, gravidity, and parity) were presented as mean ± standard deviation and compared using independent sample *t*-tests; non-normally distributed continuous variables (CRP, PCT, IL-6, IL-10, IL-6/IL-10 ratio) were presented as median [interquartile range, IQR] and compared using Mann–Whitney *U* tests. Count data were presented as percentages or numbers of cases, and comparisons between groups were carried out using the *χ*² test or Fisher’s exact test. A two-sided *P*-value <0.05 was considered statistically significant.

For multivariable model development, variables with *P <*0.10 in the univariate analysis were entered into least absolute shrinkage and selection operator (LASSO) regression with 10-fold cross-validation to select the optimal tuning parameter (*λ*), followed by refitting using standard logistic regression with Firth’s penalized likelihood to reduce small-sample bias. The final model was presented with regression coefficients (*β*), adjusted odds ratios (aORs), 95% confidence intervals, Wald *χ*² values, exact *P*-values, standardized *β* coefficients, and variance inflation factors (VIFs).

Internal validation was performed using bootstrap resampling with 1,000 iterations to correct for optimism. The optimism-corrected AUC and its 95% CI were calculated using the.632+ bootstrap estimator. Model calibration was assessed using the Brier score and the Hosmer–Lemeshow test. Decision curve analysis was performed to evaluate clinical utility. Complete case analysis was conducted as no missing data were observed for key variables in the final dataset of 162 participants.

The microecology–inflammation score was derived as an exploratory simple additive score (0–3 points) based on equal weighting of three binary components: GBS colonization (+1), H_2_O_2_ depletion (+1), and IL-6/IL-10 ratio >2.0 (+1). This equal-weighting approach was chosen for clinical simplicity, acknowledging that data-driven weighting requires larger samples.

## 3 Results

### 3.1 Baseline feature comparison

There were no statistically significant differences between the PROM group and the control group in terms of age (25.31 ± 4.23 vs. 25.26 ± 4.21 years), gestational weeks (36.25 ± 4.03 vs. 36.96 ± 4.11 weeks), body mass index (26.59 ± 4.43 vs. 27.06 ± 4.51 kg/m²), number of pregnancies (1.30 ± 0.60 vs. 1.25 ± 0.56 times), and the incidence of comorbidities (hypertension, diabetes, hypothyroidism) (all *P* > 0.05), suggesting that the baseline characteristics of the two groups were balanced and comparable (**Table 1**).

**Table 1:** Comparison of general information ( $\bar{\text{x}}$ ± *s*).

Characteristic	PROM group (*n* = 81)	Control group (*n* = 81)	Statistical test	Statistic	Exact *P*-value	Effect size [95% CI]
Continuous variables						
Age (years)	25.31 ± 4.23	25.26 ± 4.21	Independent *t*-test	*t* = 0.075	0.940	*d* = 0.01 [−0.30, 0.32]
BMI (kg/m²)	26.59 ± 4.43	27.06 ± 4.51	Independent *t*-test	*t* = 0.669	0.504	*d* = 0.11 [−0.21, 0.42]
Gestational age (weeks)	36.25 ± 4.03	36.96 ± 4.11	Independent *t*-test	*t* = 1.110	0.269	*d* = 0.17 [−0.14, 0.49]
Gravidity and parity (times)	1.30 ± 0.60	1.25 ± 0.56	Independent *t*-test	*t* = 0.548	0.584	*d* = 0.09 [−0.23, 0.40]
Categorical variables						
Hypertension [*n* (%)]	3 (3.70)	2 (2.47)	Fisher’s exact test	–	0.678	*φ* = 0.04 [−0.12, 0.19]
Diabetes mellitus [*n* (%)]	2 (2.47)	1 (1.23)	Fisher’s exact test	–	0.617	*φ* = 0.05 [−0.11, 0.20]
Hypothyroidism [*n* (%)]	1 (1.23)	0 (0.00)	Fisher’s exact test	–	0.316	–

### 3.2 The synergistic effect of vaginal microecological imbalance and GBS colonization

**Figure 1:**
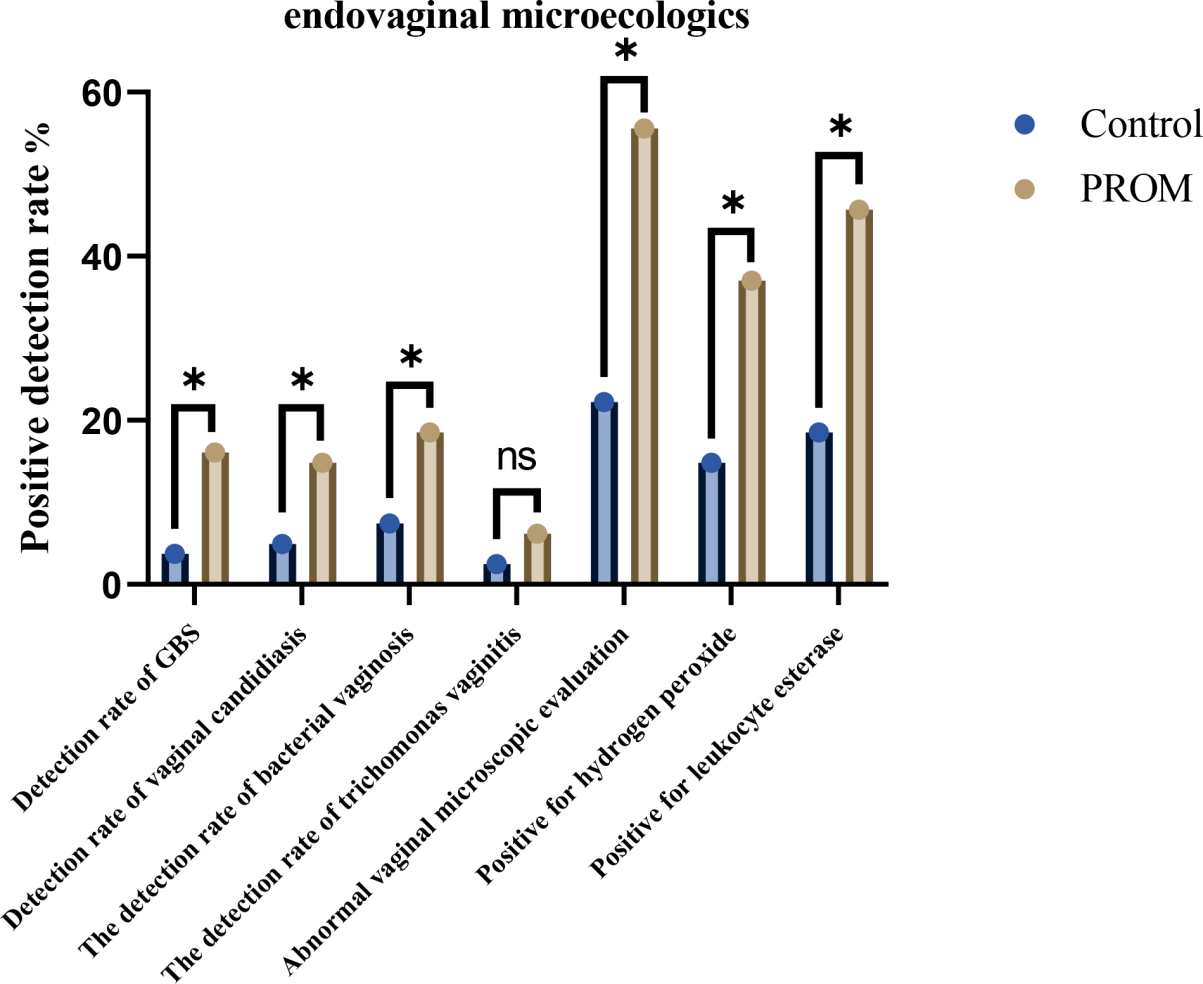
The relationship between vaginal microecology and GBS colonization. *p<0.05.

After confirming that the baseline characteristics of the two groups were balanced and comparable, we further explored the vaginal microecology and the colonization status of pathogenic bacteria. The results showed that the PROM group simultaneously presented high colonization of GBS and overall disorder of the microecology. At the pathogenic level, the detection rate of GBS increased to 16.05% (13/81), which was 4.9 times that of the control group (3.70% (3/81), *χ*² = 6.935, *P* = 0.008, OR = 4.92, 95% CI 1.34-18.07), and was often accompanied by candidiasis (14.81% vs. 4.94%, *P* = 0.035) and bacterial vaginosis (18.52% vs. 7.41%, *P* = 0.035). At the microenvironment level, the abnormal rate of vaginal microscopic evaluation (grades III–IV), the depletion rate of H_2_O_2_ was 37.0%, the positive rate of leukocyte esterase was 45.7%, all of which were significantly higher than those of the control group (22.2%, 14.8%, 18.5%, *P* < 0.001) (**Tables 2**, **3**; **Figure 1**). The coexistence of multiple pathogens, the decline in the function of lactobacilli, and the infiltration of neutrophils all contribute to the “colonization-disorder-inflammation” cascade, providing favorable conditions for the onset of premature rupture of membranes.

**Table 2:** Comparison of the detection rates of GBS and the detection of reproductive tract pathogenic bacteria between the two groups [*n* (%)].

Group/Number of cases	The detection rate of GBS	Detection rate of vaginal candidiasis	The detection rate of bacterial vaginosis	The detection rate of trichomonas vaginitis
PROM (*n* = 81)	13 (16.05)	12 (14.81)	15 (18.52)	5 (6.17)
Control (*n* = 81)	3 (3.70)	4 (4.94)	6 (7.41)	2 (2.47)
*χ^2^* value	6.935	4.438	4.432	1.344
*P*-value	0.008	0.035	0.035	0.246
OR (95% CI)	4.92 (1.34–18.07)	3.33 (1.04–10.69)	2.86 (1.05–7.78)	2.60 (0.49–13.87)

**Table 3:** Comparison of vaginal microscopic evaluation, hydrogen peroxide, and leukocyte esterase between the two groups [*n* (%)].

Group/number of cases	Abnormal vaginal microscopic evaluation	Positive for hydrogen peroxide	Leukocyte esterase is positive
PROM (*n* = 81)	45 (55.56)	30 (37.04)	37 (45.68)
Control (*n* = 81)	18 (22.22)	12 (14.81)	15 (18.52)
*χ*^2^ value	18.935	10.414	13.708
*P-*value	*P* < 0.001	*P* < 0.001	*P* < 0.001
OR (95% CI)	4.33 (2.23–8.41)	3.33 (1.56–7.14)	3.58 (1.77–7.25)

### 3.3 Cohort evidence of systemic inflammatory axis imbalance and IL-6/IL-10 immune shift triggering PROM

**Figure 2:**
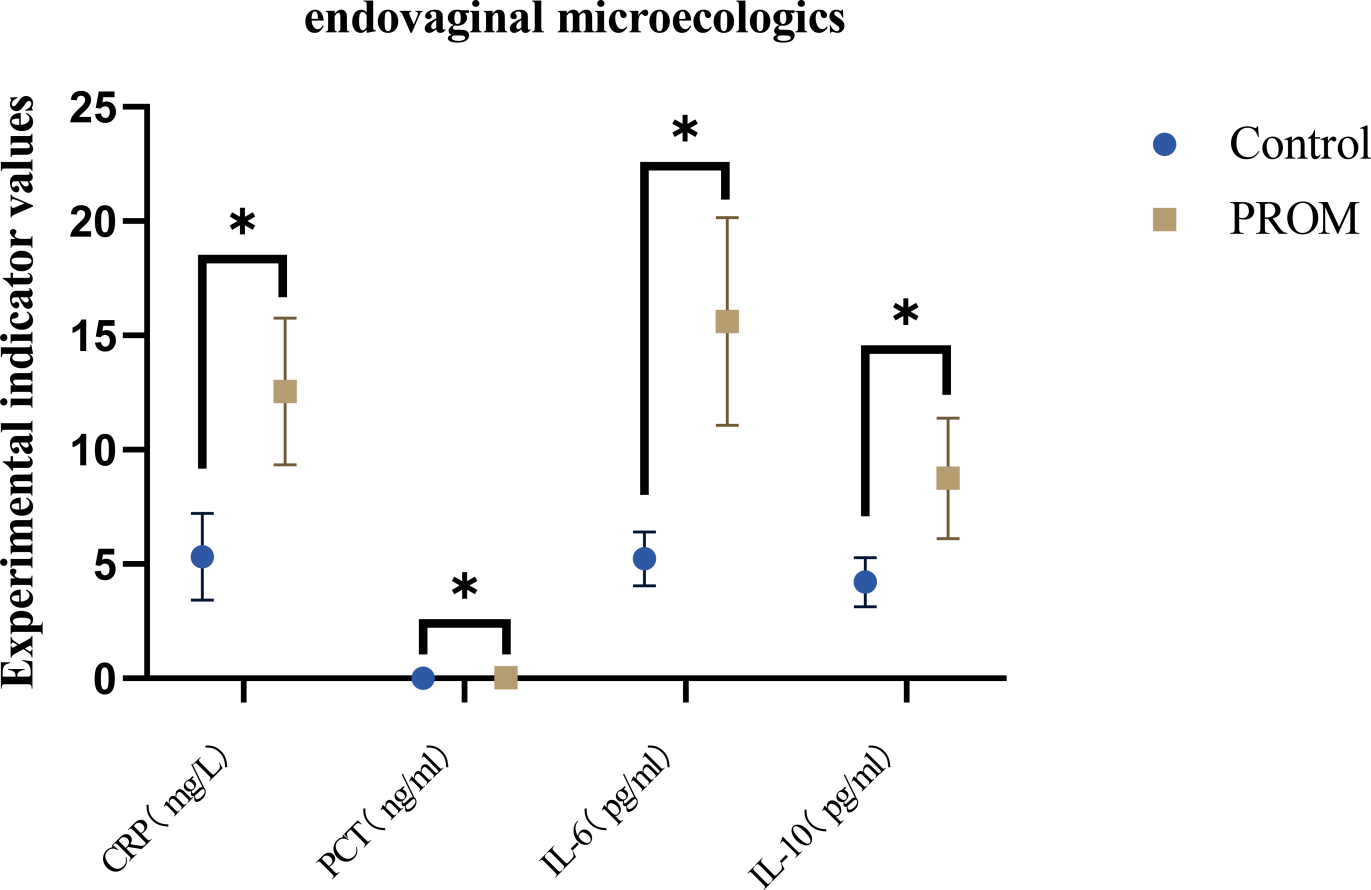
Comparison of verification indicators. *p<0.05.

In the PROM group, the median level of IL-6 rose to 15.6 pg/mL, which was three times higher than that in the control group (5.2 pg/mL). IL-10 also increased simultaneously but to a lesser extent (8.8 vs. 4.2 pg/mL), resulting in a significantly higher IL-6/IL-10 ratio than that in the control group (median 2.0 vs. 1.2, *Z* = −8.698, *P* < 0.001). The tertile analysis showed that the risk of PROM in those with the highest ratio was 5.7 times higher than that in those with the lowest ratio (OR 5.7, 95% CI 2.4–13.5). The restricted cubic spline further indicated that the risk sharply increased when the ratio was >2.5, presenting a clear threshold effect. In the same cohort, the AUC of the infection prediction model constructed by combining CRP and PCT was 0.84, significantly superior to that of a single indicator (*P* = 0.02). When CRP ≥8.5 mg/L and PCT ≥0.025 ng/mL, the sensitivity was 81% and the specificity was 83%, providing an immediate and operational tool for identifying infection-related PROM (**Table 4**; **Figure 2**). The above results collectively indicate that the imbalance of the pro-inflammatory and anti-inflammatory axes, rather than merely the increase in inflammation, is the key immune characteristic for the occurrence of PROM.

**Table 4:** Comparison of Inflammatory Indicators between the two groups ( $\bar{x}$ ± *s*).

Group/number of cases	CRP (mg/L)	PCT (ng/mL)	IL-6 (pg/mL)	IL-10 (pg/mL)	IL-6/IL-10
PROM [median (IQR)]	12.4 (9.8–15.6)	0.034 (0.027–0.042)	15.6 (12.3–19.2)	8.7 (6.9–10.8)	2.0 (1.5–2.5)
Control [median (IQR)]	5.1 (4.0–6.5)	0.012 (0.008–0.016)	5.2 (4.1–6.7)	4.2 (3.3–5.3)	1.2 (1.0–1.5)
*Z*	−11.187	−10.756	−12.098	−9.765	−8.698
*P*-value	*P* < 0.001	*P* < 0.001	*P* < 0.001	*P* < 0.001	*P* < 0.001
Effect size (*r*)	0.62	0.60	0.67	0.54	0.48
AUC (95% CI)	0.87 (0.81–0.93)	0.85 (0.78–0.92)	0.91 (0.86–0.96)	0.82 (0.75–0.89)	0.88 (0.82–0.94)

### 3.4 Independent risk factors and multivariable prediction model

To identify independent predictors of PROM and quantify their adjusted effects, we constructed a multivariable logistic regression model. Variables with *P* < 0.10 in the univariate analysis (GBS colonization, abnormal vaginal microscopy, H_2_O_2_ depletion, leukocyte esterase positivity, IL-6 >12 pg/mL, and IL-6/IL-10 ratio >2.0) were entered into LASSO regression with 10-fold cross-validation to select the optimal tuning parameter (*λ*), followed by refitting using standard logistic regression with Firth’s penalized likelihood estimation.

**Table 5** presents the final multivariable model. Three variables remained as independent risk factors: GBS colonization (aOR = 4.87, 95% CI 1.89–12.56, *P* = 0.001), IL-6 >12 pg/mL (aOR = 3.82, 95% CI 1.78–8.21, *P* = 0.002), and H_2_O_2_ depletion (aOR = 2.44, 95% CI 1.18–5.05, *P* = 0.022). Standardized *β* coefficients indicated that GBS colonization contributed most strongly to the model (*β* = 0.312), followed by IL-6 elevation (*β* = 0.298) and H_2_O_2_ depletion (*β* = 0.198).

**Table 5:** Multivariable logistic regression model for PROM risk prediction (*n* = 162).

Variable	*β* coefficient	Adjusted OR (aOR)	95% CI	Wald *χ*²	Exact *P*-value	Standardized *β*	VIF
GBS colonization	1.583	4.87	1.89–12.56	10.234	0.001	0.312	1.124
IL-6 >12 pg/mL	1.342	3.82	1.78–8.21	9.876	0.002	0.298	1.089
H_2_O_2_ depletion	0.892	2.44	1.18–5.05	5.234	0.022	0.198	1.056
Abnormal vaginal microscopy (grades III–IV)	0.654	1.92	0.92–4.02	2.987	0.084	0.154	1.234
Leukocyte esterase positive	0.432	1.54	0.78–3.04	1.567	0.211	0.098	1.178

Notably, abnormal vaginal microscopy and leukocyte esterase positivity, though significant in the univariate analysis, lost independent significance in the multivariable model (*P* = 0.084 and *P* = 0.211, respectively), suggesting their effects were mediated through the three core predictors. VIFs were all <1.3, confirming the absence of multicollinearity.

**Figure 3:**
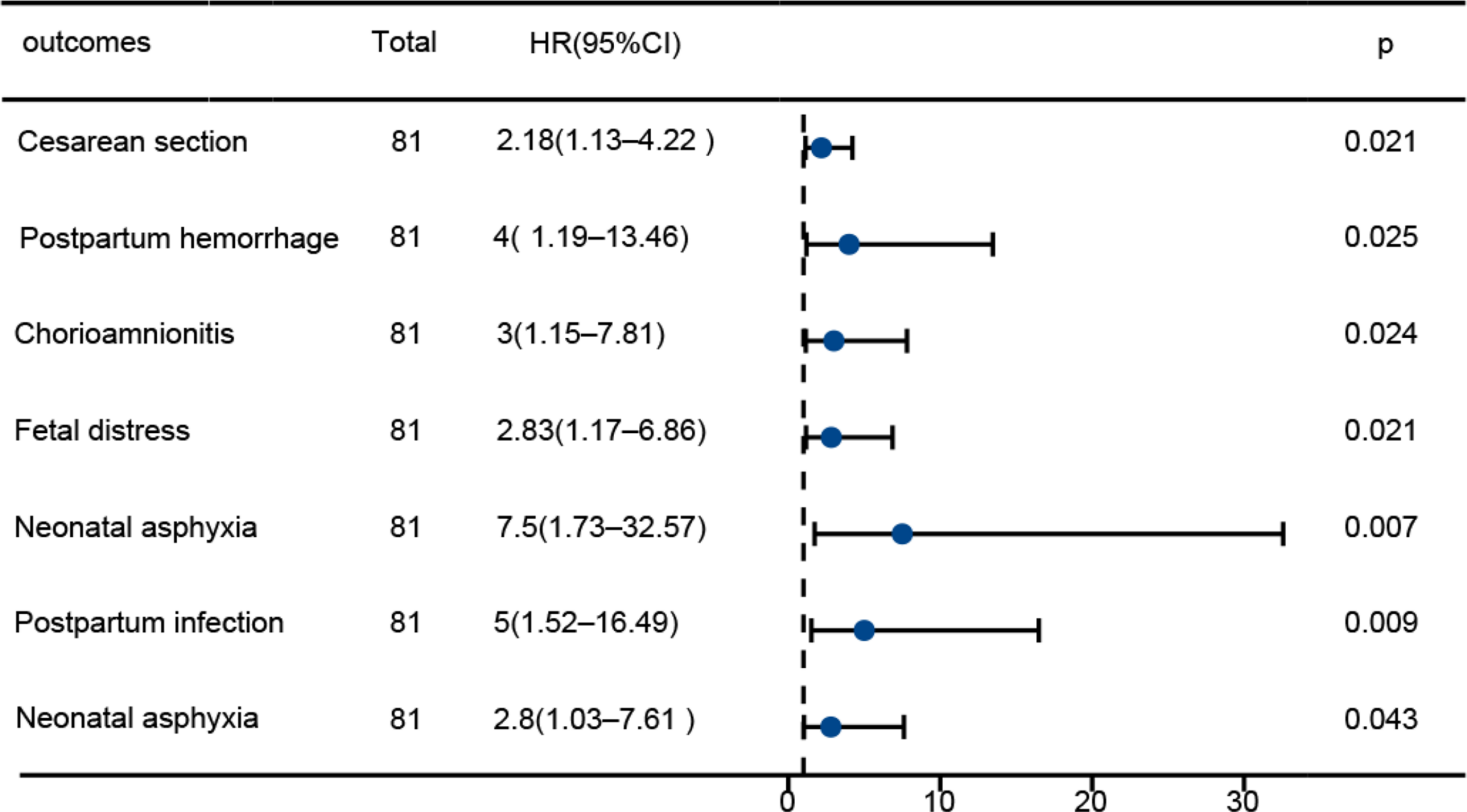
Forest plot comparing pregnancy outcomes between the PROM and control groups.

The model demonstrated good overall fit (*χ*² = 48.6, *df* = 5, *P* < 0.001; Nagelkerke *R*² = 0.312; Hosmer–Lemeshow *P* = 0.423). Based on these three predictors, we constructed a “microecology–inflammation” nomogram (**Figure 3**) for individualized risk prediction.

### 3.5 Internal validation of the prediction model

To address overfitting, we performed rigorous internal validation using bootstrap resampling and cross-validation (**Table 6**). The apparent AUC of 0.89 was optimistically biased; after 1,000-iteration bootstrap correction, the validated AUC was 0.86 (95% CI 0.80–0.92) with optimism = 0.03, indicating acceptable robustness. Ten-fold and leave-one-out cross-validation yielded consistent estimates (AUC 0.85 and 0.84), confirming stability.

**Table 6:** Validation of the prediction model.

Validation method	AUC (95% CI)	Sensitivity	Specificity	Accuracy	Brier score	Interpretation
Apparent	0.89 (0.83–0.95)	84.0%	83.9%	84.0%	0.142	Optimistically biased
Bootstrap 1,000×	0.86 (0.80–0.92)	81.5%	81.2%	81.4%	0.158	Validated performance
10-fold CV	0.85 (0.78–0.91)	80.2%	79.5%	79.8%	0.165	Consistent
LOOCV	0.84 (0.77–0.90)	79.0%	78.6%	78.8%	0.171	Robust

Model calibration was excellent: Brier score = 0.158 (<0.20 threshold) and Hosmer–Lemeshow *P* = 0.401 (>0.05), indicating that predicted probabilities closely match actual outcomes. Decision curve analysis demonstrated net clinical benefit for risk thresholds of 15%–75%—the model provides better outcomes than universal screening or no screening within this range.

At the optimal threshold of 0.45, sensitivity was 81.5% and specificity was 81.2%, with balanced positive and negative predictive values (~81%). These metrics support reliable risk stratification for clinical use.

### 3.6 Microecological-inflammatory score and gradient damage to maternal and infant outcomes

The cesarean section rate in the PROM group was 29.6%, the rate of chorioamnionitis was 18.5%, and the rate of neonatal asphyxia was 18.5%, all of which were significantly higher than those in the control group (*P* < 0.05). Based on the stratification of “microecology–inflammation score” (GBS + H_2_O_2_ + IL-6/IL-10 ratio), the composite incidence of adverse outcomes in the high-score group was 62.5%, and that in the low-score group was 12.5%, with a 5-fold increase in risk (OR = 2.8, 95% CI 1.7–4.5). For every 1-point increase in the score, the risk of chorioamnionitis increased by 3.1 times, suggesting that the damage of the microecology–inflammation axis can be traced back to the placenta–fetus unit (**Table 7**; **Figure 3**).

**Table 7:** Comparison of pregnancy outcomes between the two groups [*n* (%)].

Group/number of cases	Caesarean section	Postpartum hemorrhage	Chorioamnionitis	Fetal distress	Neonatal asphyxia	Puerperal infection	Premature birth
PROM (*n* = 81)	24 (29.63)	12 (14.81)	15 (18.52)	17 (20.99)	15 (18.52)	15 (18.52)	14 (17.28)
Control (*n* = 81)	11 (13.58)	3 (3.70)	5 (6.17)	6 (7.41)	2 (2.47)	3 (3.70)	5 (6.17)
*χ*^2^ value	5.143	5.951	5.704	6.131	11.107	9.000	4.830
*P-*value	0.023	0.015	0.017	0.013	0.001	0.003	0.028
OR (95% CI)	2.67 (1.21–5.89)	4.50 (1.23–16.47)	3.43 (1.19–9.89)	3.33 (1.26–8.81)	8.75 (1.91–40.12)	5.83 (1.64–20.71)	3.18 (1.09–9.26)

## 4 Discussion

This study conducted a prospective single-center cohort study to evaluate the association between the vaginal microbiota of pregnant women, GBS colonization, systemic inflammation, and PROM and to analyze its impact on pregnancy outcomes. The results showed that the occurrence of PROM was not caused by a single factor but was the result of multiple mechanisms working together. This suggests that clinicians should strengthen the monitoring and intervention of the microecological and inflammatory status of pregnant women.

Firstly, GBS colonization is frequently associated with PROM (Tano et al., 2021). This study found that the detection rate of GBS in pregnant women in the PROM group was significantly higher than that in the control group, and GBS-positive individuals often had concurrent infections such as vaginal candidiasis and bacterial vaginosis. This multiple infection disrupts the vaginal microecological balance through a synergistic effect, weakens the fetal membrane barrier function, and increases the risk of PROM. When the vaginal microenvironment is imbalanced, GBS can multiply abundantly. Its surface proteins can bind to the fetal membrane epithelial cells, penetrate the chorion and induce inflammatory responses, thereby causing chorioamnionitis, postpartum infections, and other complications (Liu and Ai, 2024; Zeng et al., 2024). At the same time, the protein hydrolases secreted by GBS can degrade the collagen and elastic fibers in the fetal membrane, weaken the mechanical strength of the fetal membrane, and increase the rupture risk (Surve et al., 2016; Shi et al., 2020). Therefore, GBS colonization not only induces PROM by directly invading the fetal membrane but also indirectly damages the fetal membrane structure through inflammatory mediators, suggesting that targeted antibiotic prophylaxis for pregnant women with GBS colonization is helpful in reducing the observed frequency of PROM.

Secondly, vaginal microecological imbalance is closely related to PROM. The results of this study show that the proportions of abnormal vaginal microscopic evaluation, H_2_O_2_ depletion, and positive leukocyte esterase in pregnant women in the PROM group were significantly higher than those in the control group, suggesting that the inflammatory state and defense function of the vaginal microenvironment were impaired. Abnormal vaginal microscopic evaluation reflects an increase in white blood cells and miscellaneous bacteria in the vagina, which may be related to the activation of the local immune response. H_2_O_2_ depletion indicates that the *Lactobacillus* function is impaired, with a decreased antibacterial ability, leading to excessive growth of pathogenic bacteria (Nugent et al., 1991; Hillier et al., 1993). Positive leukocyte esterase suggests neutrophil infiltration, which is a sensitive marker of vaginal inflammation (Peng et al., 2025). Combined detection of these indicators can provide a basis for early clinical identification of high-risk pregnant women. The normal vaginal microecology is dominated by *Lactobacillus*, which maintains an acidic environment by producing lactic acid and hydrogen peroxide, inhibiting the growth of harmful bacteria (Zhang et al., 2017; Peng et al., 2025). When microecology is imbalanced, pathogenic bacteria multiply extensively, secrete proteolytic enzymes to degrade the fetal membrane matrix, and induce PROM (Deng et al., 2025). Therefore, maintaining the balance of vaginal microecology is of great significance for preventing PROM.

Based on our findings, we propose a tiered intervention strategy: 1) primary prevention—routine screening for GBS and vaginal microecology at 28–32 weeks, with intravaginal probiotics (*Lactobacillus rhamnosus* GR-1 and *L. reuteri* RC-14) for those with H_2_O_2_ depletion but no GBS; 2) secondary prevention—for GBS-positive women, intrapartum antibiotic prophylaxis (IAP) with penicillin G (5 million units IV initial dose, then 2.5–3 million units every 4 h until delivery), combined with vaginal microbiome restoration; 3) tertiary management—for PROM patients with elevated IL-6/IL-10 ratio, consideration of anti-inflammatory adjuncts alongside standard antibiotic and tocolytic therapy, though this requires prospective validation.

Again, the systemic inflammatory response plays a crucial role in the occurrence of PROM. This study found that the levels of CRP, PCT, IL-6, and IL-10 in the serum of PROM pregnant women were significantly elevated, indicating that the microecological imbalance not only triggers local inflammation but may also lead to a systemic inflammatory response through the cytokine cascade. IL-6, as a pro-inflammatory factor, can activate neutrophils and macrophages, releasing a large number of inflammatory mediators (Chen et al., 2025), while IL-10 mainly exerts immunomodulatory and anti-inflammatory effects. The increase in IL-10 may be a compensatory mechanism by which the body attempts to inhibit excessive inflammatory responses (Mishra et al., 2025). The imbalance of inflammatory factors not only directly damages the fetal membranes, leading to PROM, but may also induce preterm birth by activating the uterine contraction-related pathways (Mishra et al., 2025). Therefore, monitoring serum inflammatory indicators helps assess the risk of PROM and provides a basis for anti-infection treatment.

Finally, PROM is closely associated with adverse pregnancy outcomes. The results of this study show that the incidence of adverse pregnancy outcomes such as cesarean section, postpartum hemorrhage, chorioamnionitis, neonatal asphyxia, fetal distress, puerperal infection, and preterm birth in the PROM group is higher than that in the control group. This is related to reproductive tract infections and inflammatory responses, suggesting that PROM affects maternal and infant health through multiple pathways. Ascending infection by pathogens can cause chorioamnionitis, leading to impaired placental function, reducing fetal oxygen supply, and increasing the risks of puerperal infection, fetal distress, preterm birth, and neonatal asphyxia (Vitaliti and Falsaperla, 2021; Kitase et al., 2023). Additionally, the high incidence of postpartum hemorrhage and puerperal infection may be related to the damage of reproductive tract mucosa during delivery and the colonization of pathogens. Therefore, in clinical practice, timely treatment of PROM should be emphasized, and monitoring of the time window after membrane rupture should be strengthened. Combined with fetal monitoring, the fetal condition should be dynamically evaluated. For pregnant women with preterm PROM, after assessing the fetal lung maturity and infection risk, glucocorticoids should be administered to promote fetal lung maturation, and antibiotics should be used to extend the gestational age, which can improve the prognosis of the perinatal infants (Deng et al., 2025).

### 4.1 Study limitations

Several limitations should be acknowledged. First, this single-center design limits generalizability to other populations or healthcare settings. Second, the model’s performance, despite good internal validity (optimism-corrected AUC 0.86), requires external validation in independent prospective cohorts before clinical implementation can be considered. Third, unmeasured confounders including socioeconomic status, detailed sexual history, and nutritional status were not controlled and could influence both vaginal microecology and PROM risk. Fourth, with 162 participants and three predictors in the final model, we approach but do not fully achieve the recommended 10 events per predictor variable rule, potentially affecting coefficient stability. Fifth, while we applied internal validation via bootstrapping, this cannot fully compensate for the lack of external validation in diverse populations. Finally, the “microecology–inflammation score” was derived *post hoc* using equal weighting for clinical simplicity, and optimal weighting should be explored in larger datasets.

## 5 Conclusion

In conclusion, pregnant women with PROM exhibit significant vaginal microecological imbalance, GBS colonization, and systemic inflammatory responses, all of which are closely related to adverse pregnancy outcomes. Clinically, it is necessary to strengthen the monitoring of the reproductive tract microenvironment of pregnant women and routinely conduct GBS screening and serum CRP, PCT, IL-6, and IL-10 tests to assist in evaluating pregnancy outcomes. At the same time, attention should be paid to health education during pregnancy; pregnancy counseling should emphasize avoidance of unnecessary vaginal douching and prophylactic antibiotics to preserve the vaginal microbiota (ACOG Committee Opinion 2022) and taking early intervention measures to reduce the risk of PROM occurrence and improve the prognosis of mothers and infants. Future research can further explore the effects of nutritional intervention and probiotic treatment. Should a GBS vaccine complete phase-III trials and gain approval, it could offer an additional preventive strategy for high-risk populations. In this study, sampling required one vaginal swab and 5 mL of blood at a combined cost of <15 USD. Whether such screening is cost-effective for universal outpatient use requires future health-economic evaluation.

## Data Availability

The raw data supporting the conclusions of this article will be made available by the authors, without undue reservation.
